# Reproductive ecology of interior least tern and piping plover in relation to Platte River hydrology and sandbar dynamics

**DOI:** 10.1002/ece3.4109

**Published:** 2018-05-02

**Authors:** Jason S. Alexander, Joel G. Jorgensen, Mary Bomberger Brown

**Affiliations:** ^1^ Department of Geology and Geophysics University of Wyoming Laramie Wyoming; ^2^ Nongame Bird Program Nebraska Game and Parks Commission Lincoln Nebraska; ^3^ Tern and Plover Conservation Partnership University of Nebraska Lincoln Nebraska

## Abstract

In a recent study, Farnsworth et al. (2017) used distributions of nest initiation dates drawn mostly from human‐created, off‐channel habitats and a model of emergent sandbar habitat to evaluate the hypothesis that least terns (*Sternula antillarum*) and piping plovers (*Charadrius melodus*) are physiologically adapted to initiate nests concurrent with the cessation of spring river flow rises on two sections of the Platte River, Nebraska. The study by Farnsworth et al. (2017) has several shortcomings which bring into question the authors’ principal assertion that interior least tern and piping plovers are not adapted to occupying and nesting on river sandbars on the Platte River system. We identify these shortcomings and provide information, which, we suggest, would change their conclusions if incorporated.

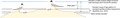

**Linked Article**: https://doi.org/10.1002/ece3.4097

## 1 INTRODUCTION

Historical and contemporary use of large, economically important rivers by threatened and/or endangered species in the United States is a subject of great interest to a wide range of stakeholders. In a recent study of the Platte River in Nebraska, Farnsworth et al. (2017) (hereinafter referred to as “the authors” or “Farnsworth et al.”) used distributions of nest initiation dates taken mostly from human‐created, off‐channel habitats and a model of emergent sandbar habitat to evaluate the hypothesis that least terns (*Sternula antillarum)* and piping plovers (*Charadrius melodus*) are physiologically adapted to initiate nests concurrent with the cessation of spring river flow rises. The authors conclude that (1) these species are not now, nor were they in the past, physiologically adapted to the hydrology of the Platte River, (2) habitats in the Platte River did not, and cannot support reproductive levels sufficient to maintain species subpopulations, (3) the gap in local elevation between peak river stage and typical sandbar height, in combination with the timing of the average spring flood, creates a physical environment which limits opportunities for successful nesting and precludes persistence by either species, and (4) the presence of off‐channel habitats, including human‐created sand and gravel mines, natural lakes, and a playa wetland, allowed the species to expand into the Platte River basin.

We suggest the authors (1) overlooked published data on the relationship between formative river stage, sandbar height, and nest heights, (2) used nest initiation dates taken from static off‐channel habitats and overemphasized the importance of mean daily hydrographs to imply that the hydrology of the Platte River system is not suitable for terns and plovers, (3) incorrectly characterized tern and plover biology, population ecology, and metapopulation dynamics, and (4) overlooked portions of the historical record which demonstrate terns and plovers were regularly present and successfully nested along the central Platte River (CPR) and lower Platte River (LPR).

## 2 FORMATIVE RIVER STAGE, EMERGENT SANDBAR HEIGHT, AND NESTING HEIGHT

**Figure 1:**
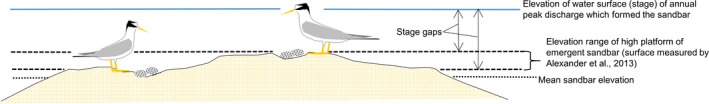
Illustration of the concept of a “stage gap” between the elevation of the top surface of an emergent sandbar and the elevation of the water surface (stage) during the annual peak discharge when the bar formed. Note that both nesting sites are on the high platform of the bar surface, but the slight topographic variation in the high platform results in different stage gaps and therefore different potential for flooding at each nesting site. Note also that the mean sandbar elevation may or may not be representative of the nesting elevation

Elevation of sandbars relative to river stage is a foundational component of the authors’ analysis as it determines whether habitat will be available or unavailable (i.e., emergent sandbars exposed above river flow level or sandbars that are fully inundated) for nesting. The sensitivity analysis presented by Farnsworth et al. (2017) showed that assumptions of sandbar heights (depth below peak river flow stage, hereafter referred to as a “stage gap”; see Figure 1 herein) accounted for the clear majority (>90%) of the variance in their emergent sandbar habitat nesting success window estimates. The authors’ stage gap assumptions and applications are problematic because of (1) the decision to not describe sandbar height data collection and analysis methods for unpublished values, (2) the assumption of a constant stage gap for each study reach despite empirical evidence to the contrary, and (3) the assumption that most nests are placed at the mean sandbar height.

The authors used mean values for the stage gap, one published (Alexander, Schultze, & Zelt, 2013) and one unpublished (the authors’ unpublished data are illustrated in their figure 7). Alexander et al. (2013) focused their height measurements on the so‐called “high platform” of emergent sandbars (see figure 3 of Alexander et al., 2013; and Figure 1 herein) rather than the entire topography of sandbars and demonstrated that their measurements overlapped with the height ranges of tern and plover nests (see figure 15 of Alexander et al., 2013). The range of sandbar heights published by Alexander et al. (2013) were shown to represent the 50th to 99th percentiles of the full sandbar topographic distribution. If the curves shown in figure 7 of Farnsworth et al. represent the full topographic distribution of sandbars in the CPR above a common reference plane, then the distributions should exclude values below approximately the median elevation value to be comparable with the Alexander et al. (2013) values. The effect of this shift would cause the mean stage gap reported in Farnsworth et al. (2017) for the CPR to decrease by about 7 to 10 cm, thereby increasing the number of years with successful nesting windows.

**Figure 2:**
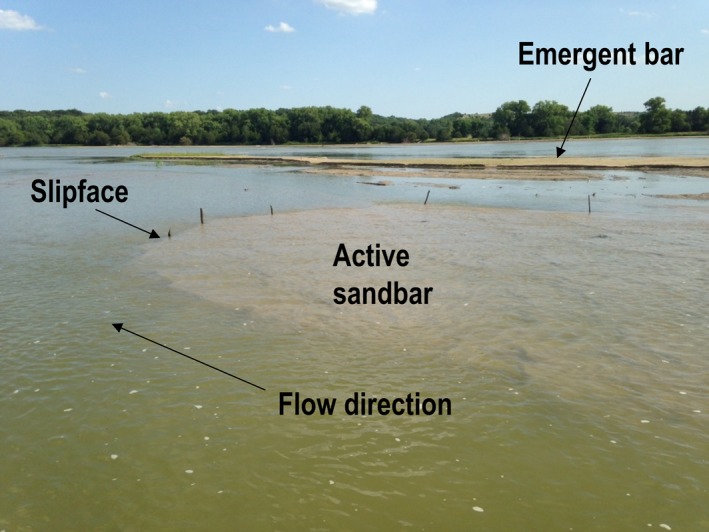
River‐level photograph of emergent and submerged (active) sandbars in the wide, braided, Niobrara River of northern Nebraska. The photograph was taken during baseflow conditions in August of 2014. The water depth over the top of the submerged sandbar in the foreground ranged from approximately 3–10 cm. The slipface of the submerged sandbar is marked by the vertical sticks. Note the flat surface of the emergent sandbar in the background; the high platform is the area above the top of the scalloped margin of the sandbar. The emergent sandbar is approximately 40–50 m long

The authors’ assumption of a constant mean value for the magnitude of the stage gap in each reach of the Platte River ignores evidence, suggesting a pattern of increasing stage gap with increasing discharge. Previous studies (Brice, 1964; Cant & Walker, 1978; Mohrig and Smith 1996; Smith 1971) indicate that sandbars submerged during low‐magnitude discharges often have shallow gaps at their crests (0.10 m or less; Figure 2). Observations of sandbars during (Ashworth et al. 2000; Crowley 1983) and following (Alexander et al., 2013) moderate‐ to high‐magnitude flow events demonstrate that the stage gap can be as much as 1 to 2 m. This concept is illustrated in figure 8 of Alexander et al. (2013), which shows that the stage gap for sandbars in the LPR formed by the 2010 flood (3,850 m^3^/s, median stage gap ~0.8 m) was much larger than the stage gap for sandbars formed by the 2011 flood (1,285 m^3^/s, median stage gap range of 0.15–0.45 m, depending on reference gage). Although Farnsworth et al. do not make clear where their value of median stage gap for the LPR was taken from, we believe the value was taken from a “group‐median” value of 2 feet (~0.61 m) reported in the summary of Alexander et al. (2013). That value was a specific statistical value (median of median sandbar heights) reported in the summary of Alexander et al. (2013) and is different than the median of the complete distribution of bar heights for the 2010 flood shown in figure 8 of that publication. Regardless, the stage gap used by Farnsworth et al. (2017) is likely associated with the much larger 2010 flood, and is between approximately 0.15 and 0.45 m larger than the stage gap reported by Alexander et al. (2013) for the more moderate 2011 flood (shown in figure 8 of that publication), and further demonstrates the need to account for variability of the stage gap with variability in discharge.

The stage gap data presented by Farnsworth et al. illustrated in figure 7 of their paper show variation in the stage gap with variable discharge, although their data generally show a decrease in median stage gap with increasing discharge (their lowest discharge created the largest median stage gap, see figure 7 in Farnsworth et al.). This odd stage gap pattern reinforces the need for an explicit description of their sandbar height data collection and analysis methods. Because of the strong control of the assumption of sandbar height on determination of successful nest windows, we suggest that Farnsworth et al. should have accounted for variation in stage gap with discharge rather than using a single value for each reach under all discharges. The larger stage gap for less frequent floods and smaller gap for more frequent floods would have the effect of increasing the number of years with successful nest windows because most years would have a smaller gap than suggested by the constant values used in each reach by Farnsworth et al.

Finally, the authors assume parity between median sandbar height and the height of nests on river sandbars, despite the fact that empirical evidence indicates (1) sandbars selected by the species for nesting tend to have mean elevations that are higher than unoccupied sandbars in the same reach and (2) nest sites selected by individual birds tend to occupy the higher regions of a sandbar's topography (see figure 1 and table 1 of Smith & Renken, 1991; tables 3 and 4 of Ziewitz, Sidle, & Dinan, 1992; table 7 of Brown and Jorgensen (2008), and figure 15b and 15c of Alexander et al., 2013). The consequence of selection of nest sites at higher elevations by the species is reduced risk of nest inundation. This concept is demonstrated in table 5 of Ziewitz et al. (1992), which shows that median and maximum nest elevations were safe from inundation in 40% and 90% of years, respectively (measurements were made in CPR and LPR, 1958–1988). As terns and plovers select higher sandbars and nest in higher locations on those sandbars, the number of years with successful nesting windows is certainly higher than those reported by Farnsworth et al. (2017).

## 3 COMPARISON OF AVERAGE PLATTE RIVER HYDROGRAPH WITH NEST INITIATION DATE DISTRIBUTIONS

In section 3.1 of Farnsworth et al., the authors use an overlay of the long‐term mean daily hydrograph (long‐term mean daily discharge values for each day of the year) for segments of the Platte River with distributions of nest initiation dates for both species (figure 8 in Farnsworth et al.) to assert that the annual spring rise typically occurs after the nest initiation date for both species. The authors use this simple overlay to suggest (see abstract, section 3.1. and discussion of that paper) that the hydrology of the Platte River creates adverse physical conditions for nesting because the typical spring rise would occur after nest establishment and, due to the large stage gaps assumed by the authors, typically inundate established nests.

Although the mean daily hydrograph can be useful for understanding basic hydrologic patterns at a location in a river, such hydrographs mask variability, particularly in the timing of the annual instantaneous peak flow, which is the typical emergent sandbar habitat formative event. For example, the mean daily hydrograph illustrated in figure 8 of Farnsworth et al. shows the late spring rise in the historical and contemporary CPR occurs in mid‐ to late June, but the peak flow record at the long‐term stream gage at the downstream end of the CPR (USGS gage no. 06774000, period of record 1896 to 2016, 13 years of missing records) indicates that 60% of annual instantaneous peaks occurred before June 1 (February through May), while 30% occurred sometime in June, and the rest at other times of the year. Although the long‐term gage is not within the Farnsworth et al. study reach (termed AHR by Farnsworth et al.), for the overlapping periods of record, more than 80% of peak flows at USGS gages within the AHR (06770000, 06770200, 06770500) are either earlier, or within 10 days of the peak flows at the long‐term gage (06774000). The peak flow records at these USGS gages on the CPR all indicate that at least 50% of peak flows occurred sometime between February 1 and May 31, and between 36% and 48% of peaks occurred before May 1. On the LPR, the peak flow record (USGS gage no. 06805500, period of record 1953–2016) indicates that 26% of instantaneous peak flows occurred before May 1 (February through April), 50% before June 1, and 30% occurred sometime in June. Farnsworth et al. account for variability in flood timing within their sandbar availability model using the daily records, but in Section 3.1 use their figure 8 to suggest a general dissonance between the timing of nest initiation and the timing of annual high flows. A more informative way to visualize and compare the general timing of nest initiation with annual peaks would have been to plot the timing and magnitude of instantaneous peaks for each reach over the nest initiation distributions. Such an overlay would inform the reader of the year‐to‐year variability in flood timing relative to the nest initiation distribution and would be a more accurate portrayal of hydrologic conditions relevant to nesting.

The authors’ distributions of nest initiation dates only include data from “all on‐channel and off‐channel” (Farnsworth et al., page 2) from the CPR for the years 2001–2013. Although not stated in their paper, nearly all (more than 96%, *n* = 1,089) of the nests reported in the CPR during this 13‐year period were found on human‐created off‐channel habitats (mostly sand and gravel mines; Baasch, 2014; Howlin, Strickland, & Derby, 2008), where suitable nesting habitat is always available when terns and plovers arrive in spring. Using nest initiation data from static, human‐created, off‐channel habitat is an incomplete representation of the species’ breeding phenology and range of nest initiation dates. This can easily result in incorrect or misleading conclusions when applied to species’ behavior in dynamic river systems where nesting habitat is not always available for nesting upon the birds’ arrival in spring. Nest initiation in many avian species (e.g., Gilbert & Servello, 2005), including terns and plovers (Elliott‐Smith & Haig, 2004; Thompson et al., 1997), is variable and occurs in response to environmental conditions. For example, least tern nest initiation on the LPR from 2008 to 2013 occurred later at river habitats (median = 16 June) compared to off‐channel habitats (median = 10 June, *t*_1,193_ = 4.97, *p* < .001; JGJ, MBB, pers. obs.). Least tern mean nest initiation dates on the Yellowstone River, Montana, where off‐channel habitats are not available, occurred 16 June, 30 June, and 1 July in 1994, 1995, and 1996, respectively, following cessation of spring rises that occurred as late as mid‐ to late June (Bacon & Rotella, 1998).

On the lower Mississippi River, which Farnsworth et al. suggest has hydrology more compatible with the species life history, least tern nest initiation (and inundation) is influenced by high flows that often extend into June or July (Dugger, Ryan, Galat, Renken, & Smith, 2002; Smith & Renken, 1993; Szell & Woodrey, 2003). Even though there may not be an extensive historical record showing nest initiation dates substantially different than what has been recently observed, as the authors state, more contemporary studies (e.g., Bacon & Rotella, 1998) do show least tern and piping plover nest initiation can be temporally variable and occur in response to variable hydrological conditions.

## 4 TERN AND PLOVER POPULATION ECOLOGY

Farnsworth et al. suggest that meeting or exceeding reproductive rates (fledge ratios) found in a report (Lutey, 2002) are necessary to maintain “stable to growing populations” of piping plovers and least terns along the Platte River. They provide calculations that purport to show the biologically improbable reproductive rates (e.g., 7.06 fledglings/pair for piping plovers) regularly needed during the years when their hydrological analysis suggests nesting was possible on the Platte River. These calculations led the authors to their principal conclusion that the historical CPR was, and contemporary LPR is, incapable of supporting least tern and piping plover populations.

The analytical approach used by the authors is too simple to address complex questions about metapopulation dynamics. Metapopulations persist as component populations that appear and disappear over space and time (Catlin et al., 2016; McGowan, Catlin, Shaffer, Gratto‐Trevor, & Aron, 2014; Zeigler et al., 2017). The authors’ calculations incorrectly assume closed populations (or that immigration and emigration are equal) within the CPR and within the LPR, which is not valid because (1) it is inconsistent with the ecology or behavior of either species and (2) does not recognize individual birds are capable of dispersing to and breeding in other locations when conditions along the Platte River are not conducive for nesting or that birds from other areas are capable of colonizing the Platte River when habitat is available. Observations of increasing local populations of least terns in areas where reproductive rates (<0.51 fledglings per pair) were well below the rates used by the authors (0.70 fledglings per pair) underscore the limitations of not considering all aspects of the species ecology when addressing questions of local population persistence (Kirsch & Sidle, 1999).

Piping plovers and least terns are capable of dispersing widely and occupying nesting habitats over broad spatial scales (Catlin et al., 2016; Elliott‐Smith & Haig, 2004; Hunt et al., 2015; Roche, Gratto‐Trevor, Goossen, & White, 2012; Roche et al., 2016; Thompson et al., 1997; Ziegler et al. 2017). Both species are relatively long‐lived and can experience high reproductive success and high reproductive failure (Elliott‐Smith & Haig, 2004; Thompson et al., 1997). These are significant aspects of both species’ life history strategies that allow them to occupy and persist in dynamic environments. Both species will renest if their nests fail during early stages of incubation (Elliott‐Smith & Haig, 2004; Thompson et al., 1997), and both species can maintain viable populations without annual breeding, breeding successfully, or achieving a certain reproductive rate at all sites or in arbitrarily defined river segments (Catlin et al., 2016; Lott, Wiley, Fischer, Hartfield, & Scott, 2013; McGowan et al., 2014). Piping plovers are known to successfully breed in one area, disperse long distances, and breed again within the same nesting season (Hunt et al., 2015). Birds occupying new or replenished habitats may experience reproductive success followed by declines in local populations and reproduction as habitat quality declines (Catlin et al., 2016; Cohen, Houghton, & Fraser, 2010). A more germane question about the terns and plovers that nested on the historical, and which continue to nest on the contemporary Platte River, is how those birds interacted, and interact, with other regional populations of their species’ metapopulation. Successful nesting occurred, and until recently (late 20th century) still occurred, on in‐channel habitats in the historical CPR and still occurs on in‐channel habitats in the contemporary LPR. These habitats contributed to, and still do contribute, to the overall metapopulation of both species in the midcontinent of North America.

## 5 HISTORICAL RECORD

The authors expressed doubts about the historical occurrence of least terns and piping plovers nesting on in‐channel (sandbars) habitat of the Platte River and suggest human‐created off‐channel habitats were both species’ primary nesting habitat which allowed them to “expand into and persist in a basin where hydrology is not ideally suited to their reproductive ecology (Farnsworth et al., pages 9–10).” To support their contentions, the authors refer only to 20th‐century nesting on sandbars and human‐created habitats along the CPR and off‐channel nesting by least terns during 2 years at a single playa wetland in the Rainwater Basin of south‐central Nebraska and along lake shorelines.

A more rigorous review of the historical record shows that least terns and piping plovers were found along the Platte and other regional rivers since the earliest recorded ornithological observations. Lewis and Clark observed least terns and piping plovers along the Missouri River in 1803–1804, as did numerous others during the late 1800s and early 1900s (Catlin et al., 2010). Least terns were observed at the Platte–Missouri River confluence in 1823 (Ducey, 2000). The earliest observation of piping plovers on the Platte River occurred on 8 July 1857 when members of the Warren Expedition collected five piping plover specimens and observed least terns at the confluence of the Loup and Platte rivers, a location 160 km upstream from the Platte–Missouri river confluence and between the two river sections considered by the authors (Ducey, 2000). Least terns were observed upstream of the historical CPR on the Platte River near the Colorado border in 1859 (Ducey, 2000).

In the first major review of Nebraska avifauna, Bruner, Wolcott, and Swenk (1904) concluded piping plovers were fairly common migrants that bred along the Platte, Loup, and Niobrara rivers and at lakes in the Sandhills of north‐central Nebraska. Bruner et al. (1904) described the least tern as a common migrant and “not a rare breeder” in Nebraska, citing nesting records along the Missouri and Niobrara rivers and at a Rainwater Basin playa wetland in 1896 and 1897 (Tout, 1902). Both species have been widely observed breeding on the Platte and other Great Plains rivers, as well as other habitats, and historically, both species were widespread and numerous. Various authors (Currier, Lingle, & VanDerwalker, 1985; National Research Council, 2005; USFWS, 2006) have concluded the Platte and other Great Plains rivers were areas of regular breeding prior to major anthropogenic modifications of the rivers. Contemporary nesting by piping plovers and/or least tern populations on other Great Plains rivers, such as the Niobrara (Adolf, Higgins, Kruse, & Pavelka, 2001), which possess similar hydrographs, and which lack off‐channel habitats, provides additional evidence contradicting the notion that adjacent off‐channel habitats are a prerequisite for these species to colonize and breed within a river segment.

## 6 MANAGEMENT AND POLICY IMPLICATIONS

The authors state that a shift in the Platte River Recovery Implementation Program's (PRRIP) activities directed toward least tern and piping plover recovery away from in‐channel habitat restoration to off‐channel habitat maintenance represents a success of adaptive management that is “unique among riverine restoration programs” (Farnsworth et al., page 10). We believe conclusions about threatened and endangered species management and recovery, as well as stewardship of natural resources, must be made considering the full spectrum of tradeoffs and consequences. Loss of habitat due to human alterations of natural systems is the principal reason regional populations of least terns and piping plovers declined, remain small compared to historical levels, and why they were listed under the Endangered Species Act and remain on the federal Endangered Species List (USFWS, 1988, 1990). It should be noted the least tern has been proposed for federal delisting based on a number of factors, including, but not limited to, conservation efforts and increasing populations in some areas (see USFWS, 2013). Industry (i.e., sand and gravel mining) in the Platte River basin has created sequences of short‐lived patches of off‐channel nesting habitat incidental to their business activities which have played a role in the population dynamics of these two species for many decades. Off‐channel tracts of habitat along, but disconnected from, the Platte River require perpetual investments of capital and maintenance to provide adequate nesting areas for terns and plovers when they are no longer being used by industry; intensive management, including native predator exclusion and control (Keldsen & Baasch, 2016), are required to achieve and maintain reproduction by the two species in these areas.

On‐channel habitats, such as those used by the birds on the historical CPR and contemporary LPR, existed or presently exist (LPR) only in resilient, dynamic river systems and are maintained by hydrological and geomorphic processes and benefit a diversity of species (Alexander et al., 2013; Currier et al., 1985). A decision to formally withdraw from river restoration and shift focus to maintaining relatively small and intensively managed tracts of off‐channel habitat in the CPR disregards consequences beyond the scope of these two species and relegates the status of least terns and piping plovers in this region to species that are conservation reliant—imperiled species whose threats can only be managed rather than eliminated (Goble, Wiens, Scott, Male, & Hall, 2012; Scott, Goble, Haines, Wiens, & Neel, 2010). Decisions to render a species conservation reliant have been questioned (Goble et al., 2012; Scott et al., 2010) because, even though species recovery goals may be achieved, populations are only maintained through perpetual human intervention. Dynamic, albeit altered, river systems such as the Platte River and others in the Great Plains, which presently maintain nesting habitat used by least terns and piping plovers, play an important role in the ongoing recovery of both species.

## 7 CONCLUSIONS

We appreciate the authors’ efforts toward modeling sandbar availability in relation to river hydrology; however, their analysis has shortcomings which limit the study's usefulness. These shortcomings, as well as incomplete characterizations of the species’ ecology and the historical record, negate the author's assertions that least tern and piping plovers are not adapted to occupying and nesting on river sandbars on the Platte River system. Decisions relegating imperiled species to conservation reliant status need to be made only after considering the full range of tradeoffs and consequences.

## CONFLICT OF INTEREST

None declared.
